# A Case of Endobronchial Aspergilloma Coexisting With Lung Adenocarcinoma

**DOI:** 10.7759/cureus.11736

**Published:** 2020-11-28

**Authors:** Halim El Hage, Leon Fagel

**Affiliations:** 1 Pulmonary and Critical Care Medicine, Holy Redeemer Hospital and Medical Center, Meadowbrook, USA; 2 Pathology, Holy Redeemer Hospital and Medical Center, Meadowbrook, USA

**Keywords:** aspergilloma, lung adenocarcinoma, bronchoscopy

## Abstract

*Aspergillus* species can cause a variety of lung diseases. Endobronchial aspergilloma is a rare clinical entity rarely encountered and often not included in the classification of *Aspergillus* lung diseases.

An 82-year-old woman presented to the outpatient setting with wheezing, shortness of breath, and productive cough. CT of the chest showed the presence of airway enlargement in a finger in glove pattern in the right upper lobe suggestive of allergic bronchopulmonary aspergillosis. Despite adequate treatment the abnormality persisted on repeat imaging. Bronchoscopy with biopsies eventually revealed the presence of hyphal elements suggestive of *Aspergillus* and poorly differentiated adenocarcinoma.

Endobronchial aspergilloma is rare and not included in the classification of *Aspergillus* lung diseases. It is thought to result from airway colonization by *Aspergillus* species. Occasionally it can obscure an underlying lung carcinoma and thus delay the diagnosis. Diagnosis is made by pathological examination of biopsy specimens. Optimal treatment is not well established.

## Introduction

*Aspergillus* is a ubiquitous fungus that can cause a variety of clinical syndromes. The disease spectrum includes invasive pulmonary aspergillosis characterized by an invasive infection that occurs in patients with severe immune system suppression, chronic necrotizing aspergillosis that affects patients with mild immunosuppression, and allergic bronchopulmonary aspergillosis (ABPA) which is a hypersensitivity reaction to the *Aspergillus* species [1]. Aspergilloma is a mass-like fungus ball and represents a non-invasive form of pulmonary aspergillosis. It occurs primarily in patients with underlying structural lung disease, especially in those with previous conditions causing lung cavities such as tuberculosis [2]. Endobronchial aspergilloma is less well described in the literature. It is the result of a non-invasive overgrowth of the *Aspergillus* species in the airway lumen [3]. We herein present an interesting case of a dual diagnosis of endobronchial aspergilloma and endobronchial adenocarcinoma.

## Case presentation

**Figure 1:**
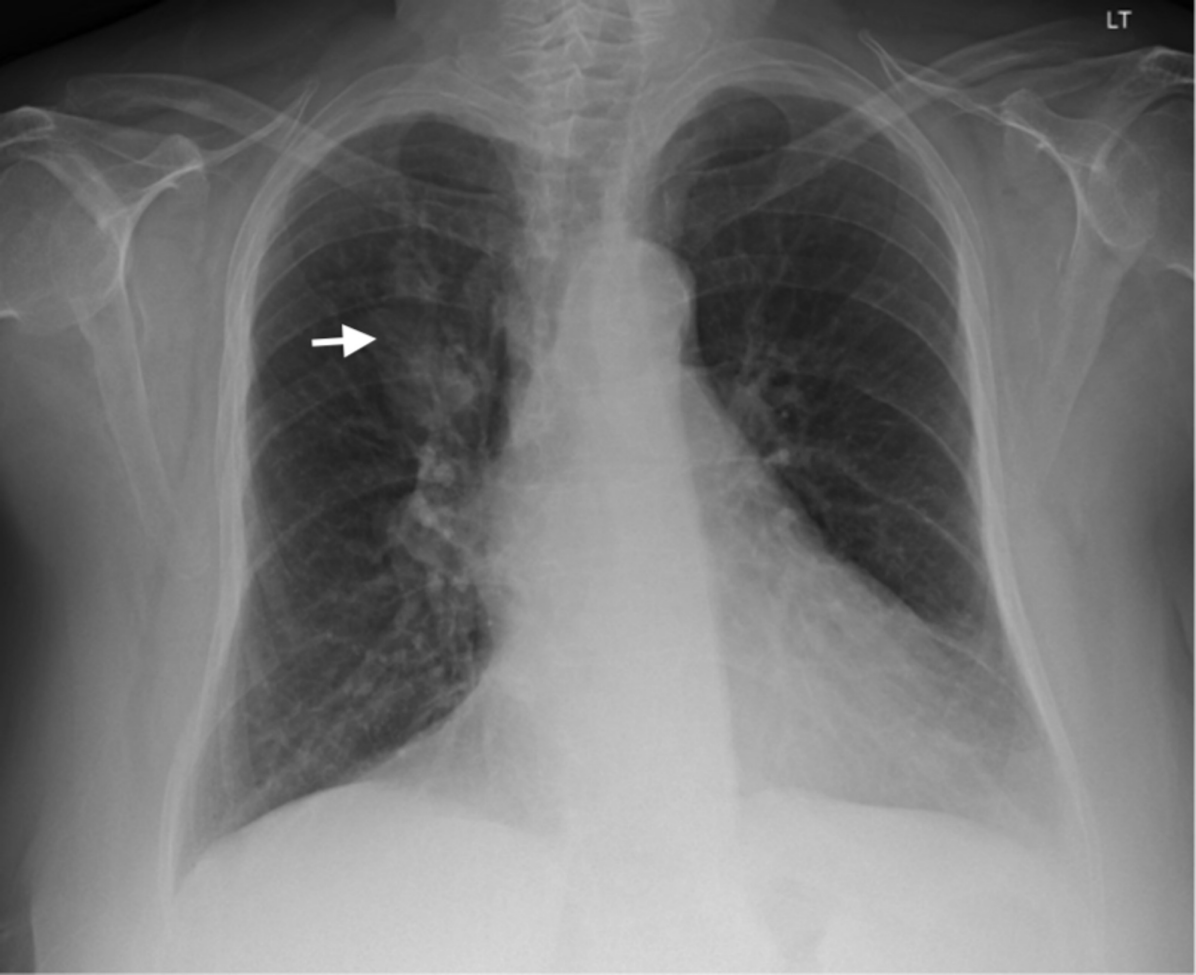
Chest Xray The image demonstrates the presence of a right upper lobe opacity (arrow).

An 82-year-old-female with a history of heavy smoking, never formally diagnosed with chronic obstructive lung disease, was referred to the outpatient pulmonary clinic for cough productive of dark yellow phlegm, wheezing, and shortness of breath that was most notable with exertion. She had a chest x-ray prior to presentation showing the presence of a right upper lobe opacity (Figure 1).

**Figure 2:**
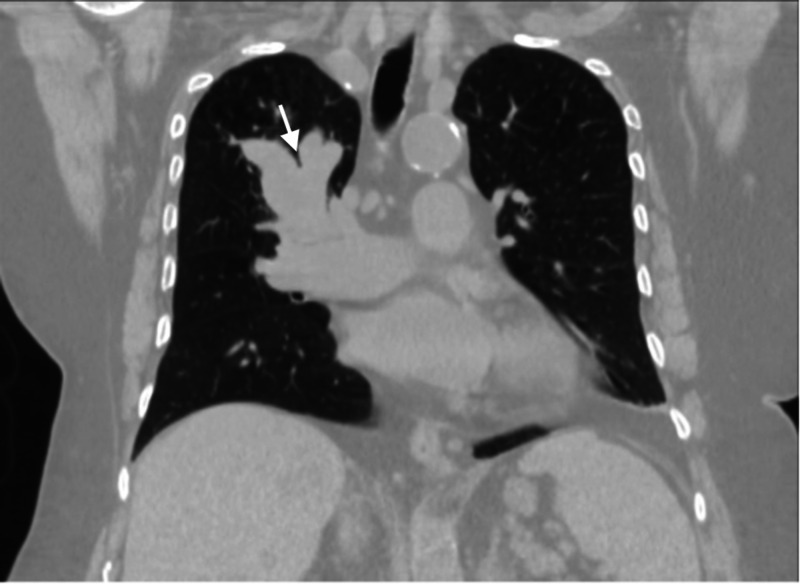
Computed tomography of the chest CT chest shows mass like enlargement of the right upper airway in a finger in glove like pattern (white arrow) suggestive of ABPA. Underlying mass cannot be excluded.

She also had computed tomography (CT) of the chest ordered by her primary care physician. The CT of the chest revealed a mass-like enlargement of the right upper lobe bronchi suggestive of ABPA (Figure 2).

**Figure 3:**
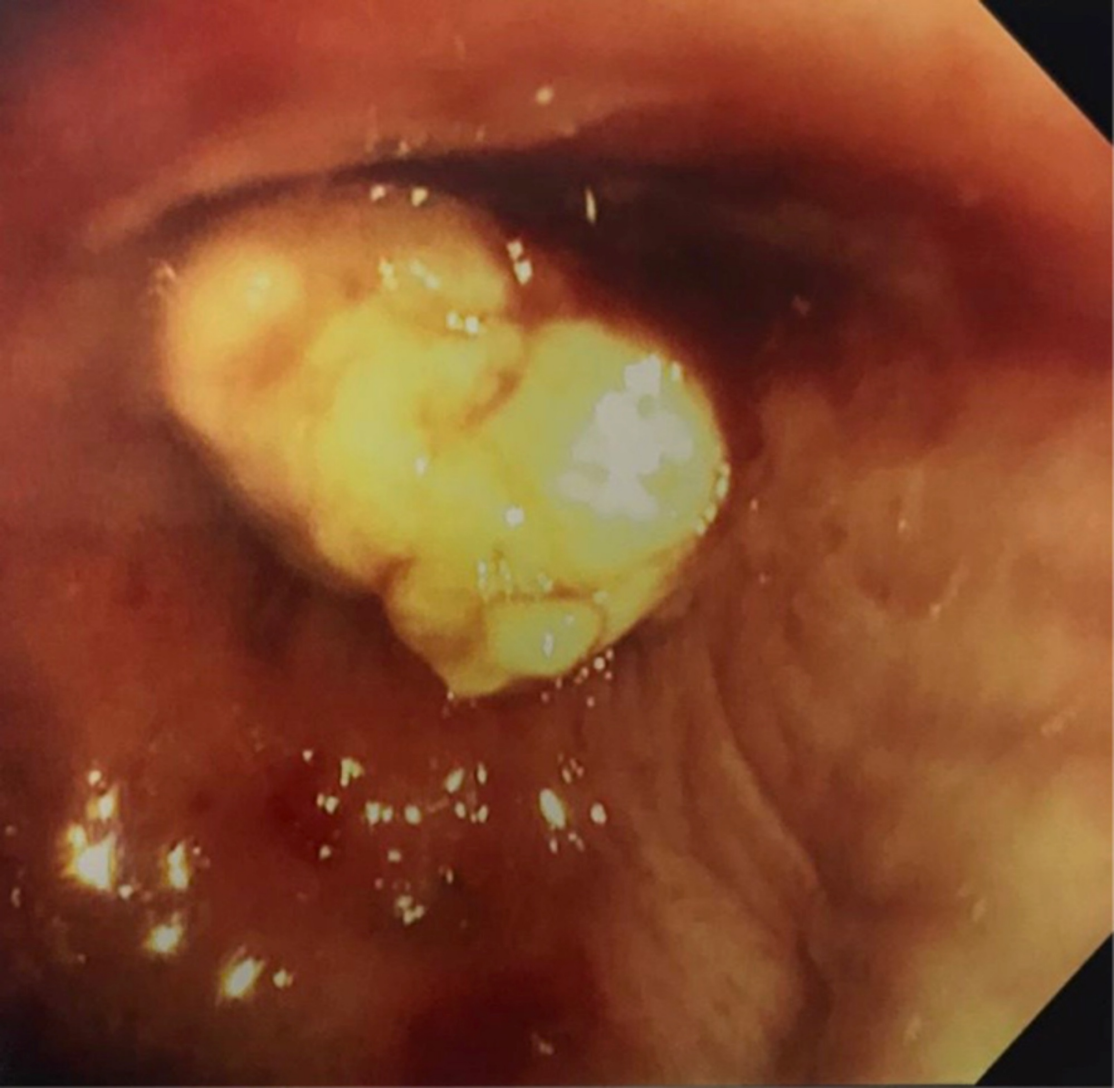
Fiberoptic Bronchoscopy Bronchoscopic examination shows a whitish mass occluding the opening of the right upper airway bronchus. Multiple biopsies were performed.

On physical examination, she was tachypneic and had diffuse expiratory wheezing. Her room air oxygen saturation was 90%. A battery of tests was ordered including complete blood count (CBC) with differential, Immunoglobulin E level (IgE), and pulmonary function tests. She was immediately started on maintenance inhalers, antibiotics, as well as systemic steroids given the gravity of her symptoms. Within a week she reported dramatic improvement in her cough, wheezing, and dyspnea. The CBC and IgE levels were within normal limits, albeit this was on systemic corticosteroids, which could have been a confounding factor. The working diagnosis at that time was ABPA. At that point, the patient was kept on a slow steroid taper, and maintenance inhalers, with plans to repeat the chest CT in a month. Clinically she was doing better. She presented a month later with improving symptoms, but her repeat CT imaging of the chest showed the persistence of the mass-like right upper lobe airway enlargement, again described as a fingers-in-glove pattern suggestive of ABPA with no evidence of enlarged mediastinal adenopathy and no contralateral lung involvement. Concerned that the opacity persisted with no reduction in size, the patient was scheduled for a bronchoscopy with possible biopsy. The procedure was done under conscious sedation and revealed the presence of a friable mass occluding the opening of the right upper lobe (Figure 3).

**Figure 4:**
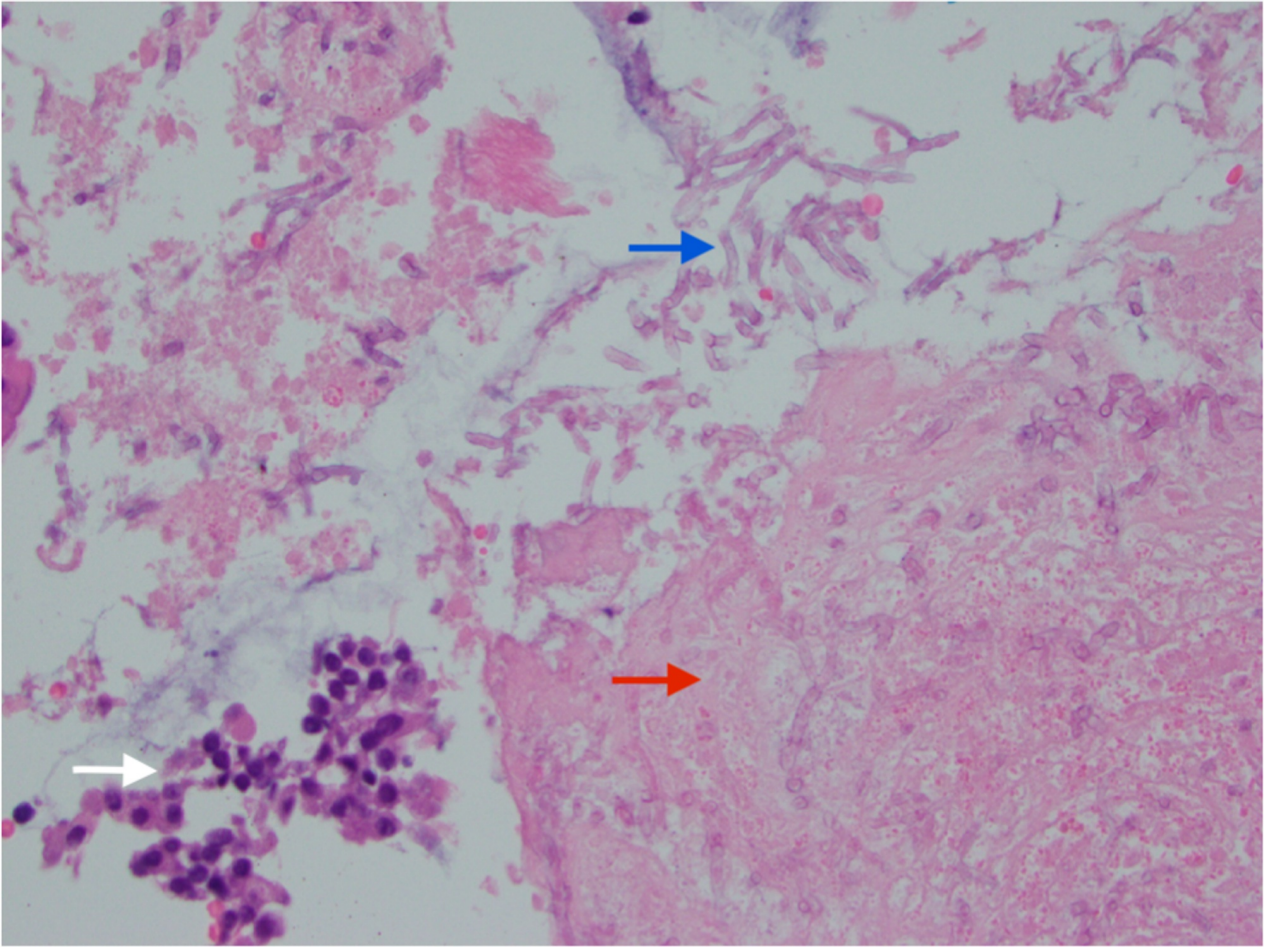
Cytopathology Right upper lobe bronchoscopic biopsy shows poorly differentiated adenocarcinoma (white arrow) with evidence of necrosis (red arrow), and hyphal elements branching at an acute angle (blue arrow).

Multiple endobronchial forceps biopsies were performed at that site. The final pathology result showed the presence of poorly differentiated adenocarcinoma with neuroendocrine features. Interestingly cytopathologic examination also revealed the presence of fungal hyphal elements and tissue necrosis (Figure 4).

The final diagnosis was endobronchial aspergilloma and poorly differentiated lung adenocarcinoma. The patient was started on oral voriconazole after consultation with the infectious disease specialist. She was then seen in a regional cancer center and staging revealed the presence of distant metastases in the liver and adrenal glands. Given her advanced age and limited functional status she opted for a palliative approach.

## Discussion

Endobronchial aspergilloma is rare disease entity not well defined in the literature. It is not usually classified with the other forms of pulmonary aspergillosis [4]. It is considered to be the unusual endobronchial form of the *Aspergillus* fungus ball and is usually limited to the main bronchi [5]. In immunocompetent patients, aspergillomas typically require structural damage to the lungs causing airway stasis which allows for endobronchial colonization by the fungus [6]. Patients usually present with cough, shortness of breath, and hemoptysis. The diagnosis is usually made with bronchoscopy and endobronchial biopsies. Bronchoscopy reveals an endobronchial white appearing mass causing airway occlusion [5]. Cytopatholigcal examination reveals the presence of hyphal elements with an acute branching angle. Lung cancer and in particular endobronchial tumors can be associated with *Aspergillus* airway colonization. A few cases of endobronchial aspergillosis coexisting with malignant lung tumors have been reported in the literature [5,7]. When lung cancer is associated with *Aspergillus* colonization, it may be obscured by endobronchial aspergilloma, and the diagnosis of lung cancer can be delayed [7,8]. It is unclear if chemotherapy presents a risk factor for recurrence of the endobronchial aspergilloma. In one case report a patient contracted invasive aspergillosis while receiving durvalumab therapy for metastatic non-small cell lung cancer [9]. The patient in that report however had several other risk factors for invasive aspergillosis. The optimal treatment of endobronchial aspergilloma is not well defined in the literature. Bronchoscopic resection coupled with systemic antifungal therapy for a period of six to 12 months can be associated with good outcomes [5]. 

## Conclusions

Endobronchial aspergillosis is a rare entity that occurs in immunocompetent patients with underlying lung disease. In rare occasions, it can obscure and delay the crucial diagnosis of lung cancer. Bronchoscopy reveals a necrotic mass causing endobronchial obstruction. Endobronchial biopsies can confirm the diagnosis and rule out any underlying malignancy. Treatment is not well defined, but bronchoscopic therapy coupled with systemic antifungals can be associated with a good prognosis. 
